# Hepatitis E Virus Infection in Dromedaries, North and East Africa, United Arab Emirates, and Pakistan, 1983–2015

**DOI:** 10.3201/eid2207.160168

**Published:** 2016-07

**Authors:** Andrea Rasche, Muhammad Saqib, Anne M. Liljander, Set Bornstein, Ali Zohaib, Stefanie Renneker, Katja Steinhagen, Renate Wernery, Mario Younan, Ilona Gluecks, Mosaad Hilali, Bakri E. Musa, Joerg Jores, Ulrich Wernery, Jan Felix Drexer, Christian Drosten, Victor Max Corman

**Affiliations:** University of Bonn Medical Centre, Bonn, Germany (A. Rasche, J.F. Drexler, C. Drosten, V.M. Corman);; University of Agriculture, Faisalabad, Pakistan (M. Saqib, A. Zohaib);; International Livestock Research Institute, Nairobi, Kenya (A.M. Liljander, J. Jores);; National Veterinary Institute, Uppsala, Sweden (S. Bornstein); EUROIMMUN AG, Lübeck, Germany (S. Renneker, K. Steinhagen);; Central Veterinary Research Laboratory, Dubai, United Arab Emirates (R. Wernery, U. Wernery);; Vétérinaires Sans Frontières Germany, Nairobi (M. Younan, I. Gluecks);; Cairo University, Giza, Egypt (M. Hilali);; Ministry of Science and Communication, Khartoum, Sudan (B.E. Musa);; University of Bern, Bern, Switzerland (J. Jores);; German Centre for Infection Research, Bonn (J.F. Drexler, C. Drosten, V.M. Corman)

**Keywords:** Hepatitis E virus, HEV, viruses, dromedaries, North Africa, East Africa, Pakistan, United Arab Emirates, zoonoses

## Abstract

A new hepatitis E virus (HEV-7) was recently found in dromedaries and 1 human from the United Arab Emirates. We screened 2,438 dromedary samples from Pakistan, the United Arab Emirates, and 4 African countries. HEV-7 is long established, diversified and geographically widespread. Dromedaries may constitute a neglected source of zoonotic HEV infections.

Hepatitis E virus (HEV) is a major cause of acute hepatitis worldwide (*1*). Four HEV genotypes belonging to the species *Orthohepevirus A* are commonly found in humans (HEV-1 through HEV-4). Genotypes 1 and 2 seem to be restricted to humans, whereas genotypes 3 and 4 also occur in domesticated and wild animals. Zoonotic transmission by ingestion of contaminated meat, mainly from pigs, is the most likely zoonotic source of infection (*1*).

Recently, HEV sequences were reported from 3 dromedaries sampled in the United Arab Emirates (UAE) in 2013 and were classified as a new orthohepevirus A genotype, HEV-7 (*2*,*3*). Afterwards a human patient also from the UAE who had chronic hepatitis after liver transplantation was shown to carry HEV-7 (*3*,*4*). Until now, knowledge on HEV-7 and its zoonotic potential relied on these 2 studies, which provide no insight into the prevalence and distribution of HEV-7. To determine the geographic distribution of HEV-7, we conducted a geographically comprehensive study of HEV-7 prevalence in dromedaries by testing 2,438 specimens sampled in 6 countries during the past 3 decades.

## The Study

**Figure 1:**
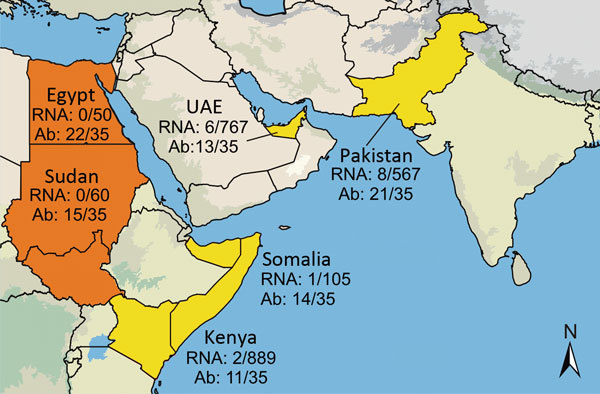
Six countries studied for hepatitis E virus (HEV) infection in dromedary camels, 1983–2015. Number of tested and number of HEV-7 RNA-positive samples or Ab-positive samples are given next to the study sites: Egypt, Sudan (today separated into Sudan and South Sudan), Kenya, Somalia, UAE, and Pakistan. Countries with both HEV-7 RNA and Ab detection are in yellow; countries with only Ab detection are in orange. Ab, antibody; UAE, United Arab Emirates; Map was created by using Quantum GIS (http://qgis.osgeo.org) and data from http://www.naturalearthdata.com.

**Figure 2:**
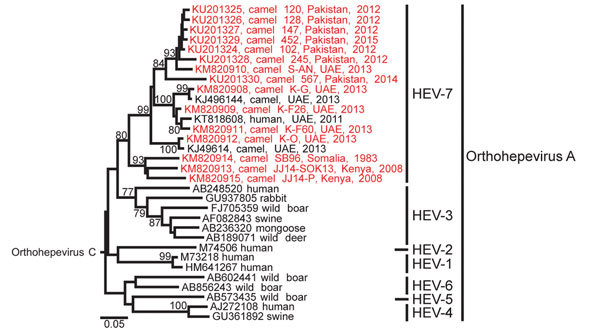
Phylogenetic analysis of *Orthohepevirus A* sequences. The analysis comprised partial hepatitis E virus (HEV) sequences (283 nt from the RNA-dependent RNA polymerase region) from this study, representatives of *Orthohepevirus A* genotypes 1–7 and *Orthohepevirus C* (GenBank accession no. GU345042) as an outgroup. The phylogenetic tree was calculated with MEGA 6.0 (http://www.megasoftware.net) by using the neighbor-joining algorithm and a nucleotide percentage distance substitution model. Bootstrap values (%) of 1,000 repetitive analyses >75 are shown next to the nodes. New camel HEV sequences obtained in this study are in red. Scale bar represents the genetic distance. All sequences obtained in this study are deposited in GenBank (accession nos. KM820907–KM820915 and KU201324–KU201330). UAE, United Arab Emirates.

Serum and fecal samples were collected from dromedary camels in the UAE, Somalia, Sudan, Egypt, Kenya, and Pakistan during 1983–2015 (*5*–*7*). A total of 2,171 serum samples and 267 fecal samples were tested for HEV RNA by using reverse transcription PCR (RT-PCR) as previously described (*8*). Seventeen samples were positive for HEV RNA: 12 (0.6%) of 2,171 serum samples and 5 (1.9%) of 267 fecal samples (Table). Positive samples originated from UAE, Somalia, Kenya, and Pakistan and dated to 1983 (Figures 1, 2**)**. Viral loads were measured by using real-time RT-PCR (*9*) calibrated on the basis of the World Health Organization International Standard for HEV RNA (*10*). Viral RNA concentrations ranged from 3.2 × 10^4^ to 3.6 × 10^7^ IU/g in feces and 6.2 × 10^2^ to 8.3 × 10^6^ IU/mL in serum.

**Table:** Sample characteristics and detection rates of orthohepevirus Agenotype 7 in 6 countries, 1983–2015

Country	Time period	No. positive/no. tested (% positive)		
		Virus RNA		Antibodies
		Serum	Feces	
Sudan	1983	0/60		15/35(42.9)
Somalia	1983–1984	1/105 (0.9)		14/35 (40.0)
Egypt	1997	0/50		22/35 (62.9)
Kenya	1992–2015	2/889 (0.2)		11/35 (31.4)
United Arab Emirates	2013	1/500 (0.2)	5/267 (1.9)	13/35 (37.1)
Pakistan	2012–2015	8/567 (1.4)		21/35 (60.0)
Total		12/2,171 (0.5)	5/267 (1.9)	96/210 (45.7)

We sequenced a 283-nt fragment of the RNA-dependent RNA polymerase gene of all positive samples for phylogenetic analyses. All camel HEV clustered in a monophyletic clade with the human HEV-7 sequence (Figure 2), supporting the classification of camel-associated HEV to a separate *Orthohepevirus A* genotype (*11*).

African viruses from Somalia and Kenya formed a monophyletic clade, whereas viruses from UAE and Pakistan were intermixed (Figure 2). Distances based on nucleotide identities were calculated for all sequences from this study and 1 reference strain from each orthohepevirus A genotype as defined by Smith et al. (*11*). This subset of references comprised GenBank accession nos. M73218 (HEV-1), M74506 (HEV-2), AF082843 (HEV-3), AJ272108 (HEV-4), AB573435 (HEV-5), AB602441 (HEV-6), and KJ496143 (HEV-7). Nucleotide diversity was remarkable among viral sequences from dromedaries, reaching a maximum distance of 22.7%, compared with a maximum distance of 29.9% among all genotypes. The internal distance among the African viruses was 14.2%, compared with 17.4% distance within viruses from UAE and Pakistan. The African viruses were 16.7%–22.7% distant from UAE and Pakistan viruses, which corresponds to the distance threshold of 22%–25% that separates the prototype HEV-4 sequence from HEV-5 and HEV-6 prototype sequences. This finding suggests that HEV-7 is a strongly diversified clade of viruses that might need to be further subclassified.

HEV-7 was recently shown to belong to the same serotype as HEV-1–4 (*12*). Therefore, we conducted a preliminary serologic analysis with a subset of 210 specimens (35 per country) by adapting a human HEV ELISA (EUROIMMUN, Lübeck, Germany) for application with camel serum. Serum was tested at a 1:100 dilution. The signal-to-noise ratio was optimized by normalizing the optical density (OD) of test samples against ODs of a reference serum included in every run (Technical Appendix Figure).

For confirmation of ELISA results and to determine an appropriate ELISA cutoff, we tested 56 samples covering the complete range of OD ratios by adapting the recomLine Immunoblot (MIKROGEN, Neuried, Germany). Thirty-two samples reacted against >2 of the presented antigens and were therefore ranked positive in the Immunoblot. All tested samples with ELISA OD ratios >0.46 were positive by immunoblot, whereas only 7 of 31 tested samples below this value were positive by immunoblot (online Technical Appendix Figure). Subsequently we set an ELISA cutoff of 0.46. Using this cutoff, we found 96 (46%) of the 210 serum samples originating from all 6 countries were positive (Table), which is comparable with the seroprevalences typically observed in pigs that are known zoonotic reservoirs for HEV-3 in developed countries (*13*). The percentage of ELISA-positive serum samples ranged from 31% in Kenya to 63% in Egypt but did not differ significantly among all 6 countries (p = 0.1, Yates’ χ^2^ test). These results suggest a wide occurrence and high prevalence of HEV in dromedaries.

## Conclusions

We investigated HEV-7 infection in dromedaries. The broad spatial extent, the high diversity of HEV-7 in dromedaries, and the detection of HEV-RNA in a sample collected in 1983 suggest a long evolutionary history of HEV-7 in dromedaries.

Our study has some limitations. First, although most tested dromedaries seemed healthy, no detailed health information from the RNA-positive animals was available. Second, we studied limited genome fragments that prevented formal classification into genome subtypes (*14*). Third, although we used 2 different antibody detection methods, the antibody prevalence in camels should be confirmed by larger studies including virus neutralization studies to determine potential genotype variability.

Investigations of camelids other than dromedaries could help to further elucidate the geographic and evolutionary origin of HEV-7. Furthermore, other wild or domestic ungulates with close contact to dromedaries could be investigated to assess the host range of HEV-7. Human infection with HEV is common in all studied areas (*1*). On the basis of clinical observations and HEV antibody detection tools, several HEV outbreaks mainly linked to water contamination or poor hygienic circumstances have been described for Pakistan, Sudan, Somalia, and Egypt. For Kenya and UAE, data about HEV prevalence is scarce (*1*). In large parts of the Middle East, human infections are unlikely to be caused by contact with swine or consumption of pork for cultural reasons. Even in Saudi Arabia, where pork is absent in diet, blood donors have antibodies at proportions of up to 18.7% (*1*). Thus, most HEV infections in the Middle East are assumed to be caused by nonzoonotic genotypes 1 and 2. However, our study and previous studies (*12*) showed that HEV-7 and other human genotypes form 1 serotype, suggesting a lack of discrimination in seroprevalence studies.

The human HEV seroprevalence in the Middle East region might in fact be caused by HEV-7 infection. Furthermore, human HEV-7 infections might contribute to the HEV prevalence in all studied areas, where camel products are frequent parts of human diet (*15*). A foodborne transmission scenario is further suggested by the fact that 1 of 12 positive serum in the study was actually sampled in a slaughterhouse, documenting that meat from infected animals can enter the food chain (*6*). Detections of HEV-7 RNA in feces in this and a previous study (*2*) point at feces or feces-contaminated camel products, such as milk, as putative additional sources of human infection. Considering the importance of dromedaries as livestock animals (*15*), risk groups, such as slaughterhouse workers, should be screened for HEV-7 infection.

Technical Appendix Individual optical density ratios obtained from ELISA testing of serum and fecal samples from dromedary camels.

## References

[1] Aggarwal R. The global prevalence of hepatitis E virus infection and susceptibility: a systematic review [cited 2016 May 16]. http://whqlibdoc.who.int/hq/2010/WHO_IVB_10.14_eng.pdf

[R2] Aggarwal R. The global prevalence of hepatitis E virus infection and susceptibility: a systematic review. In: Biologicals. Geneva: World Health Organization; 2010.

[2] Woo PC, Lau SK, Teng JL, Tsang AK, Joseph M, Wong EY, New hepatitis E virus genotype in camels, the Middle East. Emerg Infect Dis. 2014;20:1044–8. 10.3201/eid2006.140140 24856611 PMC4036782

[3] Smith DB, Simmonds P, Jameel S, Emerson SU, Harrison TJ, Meng XJ, Consensus proposals for classification of the family *Hepeviridae.* J Gen Virol. 2014;95:2223–32. 10.1099/vir.0.068429-0 24989172 PMC4165930

[4] Lee GH, Tan BH, Chi-Yuan Teo E, Lim SG, Dan YY, Wee A, Chronic infection with camelid hepatitis E virus in a liver transplant recipient who regularly consumes camel meat and milk. Gastroenterology. 2016;150:355–357.e3. 10.1053/j.gastro.2015.10.048 26551551

[5] Corman VM, Jores J, Meyer B, Younan M, Liljander A, Said MY, Antibodies against MERS coronavirus in dromedary camels, Kenya, 1992–2013. Emerg Infect Dis. 2014;20:1319–22. 10.3201/eid2008.140596 25075637 PMC4111164

[6] Müller MA, Corman VM, Jores J, Meyer B, Younan M, Liljander A, MERS coronavirus neutralizing antibodies in camels, Eastern Africa, 1983–1997. Emerg Infect Dis. 2014;20:2093–5. 10.3201/eid2012.141026 25425139 PMC4257824

[7] Meyer B, Müller MA, Corman VM, Reusken CB, Ritz D, Godeke GJ, Antibodies against MERS coronavirus in dromedary camels, United Arab Emirates, 2003 and 2013. Emerg Infect Dis. 2014;20:552–.9 10.3201/eid2004.131746 24655412 PMC3966379

[8] Drexler JF, Seelen A, Corman VM, Fumie Tateno A, Cottontail V, Melim Zerbinati R, Bats worldwide carry hepatitis E virus-related viruses that form a putative novel genus within the family *Hepeviridae.* J Virol. 2012;86:9134–47. 10.1128/JVI.00800-12 22696648 PMC3416139

[9] Girón-Callejas A, Clark G, Irving WL, McClure CP. In silico and in vitro interrogation of a widely used HEV RT-qPCR assay for detection of the species orthohepevirus A. J Virol Methods. 2015;214:25–8. 10.1016/j.jviromet.2014.11.025 25528997

[10] Baylis SA, Blümel J, Mizusawa S, Matsubayashi K, Sakata H, Okada Y, World Health Organization International Standard to harmonize assays for detection of hepatitis E virus RNA. Emerg Infect Dis. 2013;19:729–35. 10.3201/eid1905.121845 23647659 PMC3647515

[11] Smith DB, Simmonds P; International Committee on Taxonomy of Viruses Hepeviridae Study Group. Jameel S, Emerson SU, Harrison TJ, et al. Consensus proposals for classification of the family *Hepeviridae*. J Gen Virol. 2014;95(Pt 10):2223–32. 10.1099/vir.0.068429-0 PMC4165930 24989172

[12] Zhou X, Kataoka M, Liu Z, Takeda N, Wakita T, Li TC. Characterization of self-assembled virus-like particles of dromedary camel hepatitis E virus generated by recombinant baculoviruses. Virus Res. 2015;210:8–17. 10.1016/j.virusres.2015.06.022 26160190 PMC7114528

[13] Krumbholz A, Joel S, Neubert A, Dremsek P, Dürrwald R, Johne R, Age-related and regional differences in the prevalence of hepatitis E virus–specific antibodies in pigs in Germany. Vet Microbiol. 2013;167:394–402. 10.1016/j.vetmic.2013.10.001 24238666

[14] Smith DB, Simmonds P, Izopet J, Oliveira-Filho EF, Ulrich RG, Johne R, Proposed reference sequences for hepatitis E virus subtypes. J Gen Virol. 2016;97:537–42. 10.1099/jgv.0.000393 26743685 PMC5588893

[15] Kadim IT, Mahgoub O, Faye B, Farouk MM, editors. Camel meat and meat products Wallingford (UK): CAB International; 2013.

