# Correction: Vol. 22, No. 7

**DOI:** 10.3201/eid2303.C12303

**Published:** 2017-03

**Keywords:** errata, erratum, correction

The name of author Felix Drexler was misspelled in Hepatitis E Virus Infection in Dromedaries, North and East Africa, United Arab Emirates, and Pakistan, 1983–2015 (A. Rasche et al.). The article has been corrected online (https://wwwnc.cdc.gov/eid/article/22/7/16-0168_article).

