# Supplementary material for: Hepatitis E Virus Infection in Dromedaries, North and East Africa, United Arab Emirates, and Pakistan, 1983–2015
Source: Emerg Infect Dis. 2016 Jul;22(7):1249–52. doi: 10.3201/eid2207.160168 (PMC4918144; doi:10.3201/eid2207.160168)
Supplement: Technical Appendix — Individual optical density ratios obtained from ELISA testing of serum and fecal samples from dromedary camels. [file 16-0168-Techapp-s1.pdf]

Article DOI: http://dx.doi.org/10.3201/eid2207.160168

Hepatitis E Virus Infection in Dromedaries, North and East Africa, United Arab Emirates, and Pakistan, 1983–2015

Technical Appendix

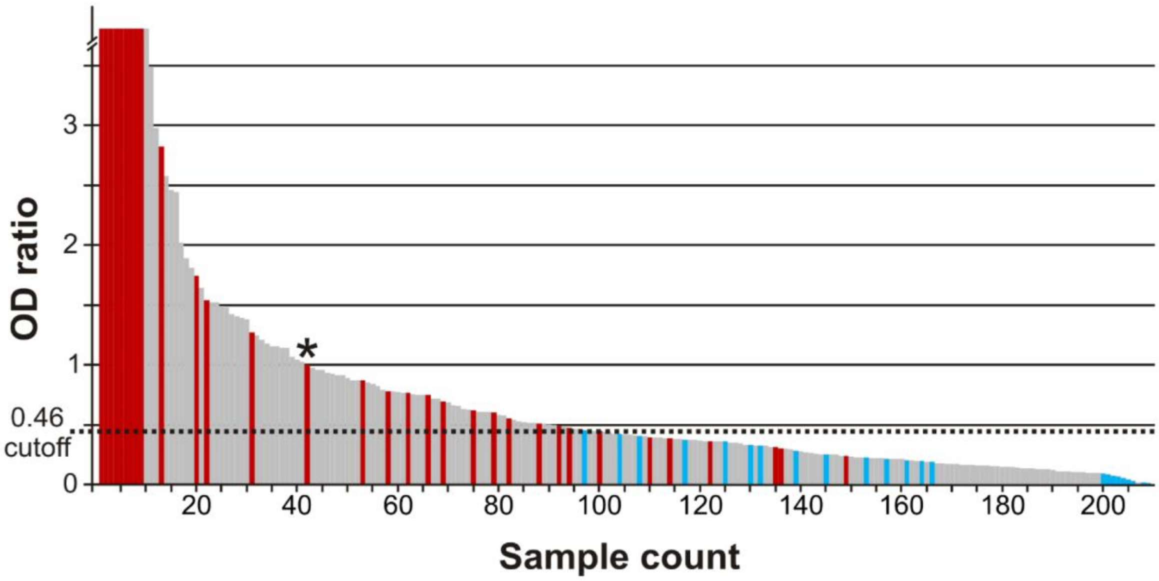

Technical Appendix Figure. Individual optical density (OD) ratios obtained from ELISA testing of serum and fecal specimens from dromedary camels for hepatitis E virus. The OD ratio was calculated by dividing the OD, measured at 450/605 nm, of each sample by the OD of a reference serum (marked with an asterisk), which was obtained from a dromedary 7 months after reverse transcription PCR–confirmed HEV infection and which was tested in parallel in all experiments. Fifty-six samples were also tested in the recomLine Immunoblot (MIKROGEN, Neuried, Germany) for confirmation. These samples were marked red (if Immunoblot testing was positive) or marked cyan (if Immunoblot testing was negative) Dashed line indicates the ELISA cutoff >0.46.
